# Elevated Salivary Theobromine and Long‐Term Improvement of Periodontal Health in Two Cohort Studies

**DOI:** 10.1111/jcpe.70072

**Published:** 2025-12-12

**Authors:** Thomas Kocher, Sebastian‐Edgar Baumeister, Henry Völzke, Matthias Nauck, Peter Meisel, Karsten Suhre, Uwe Völker, Nele Friedrich, Birte Holtfreter

**Affiliations:** ^1^ Department of Restorative Dentistry, Periodontology and Endodontology University Medicine Greifswald Greifswald Germany; ^2^ Institute of Health Services Research in Dentistry University of Münster Münster Germany; ^3^ Institute for Community Medicine University Medicine Greifswald Greifswald Germany; ^4^ German Center for Cardiovascular Research (DZHK), Partner Site Greifswald, University Medicine Greifswald Greifswald Germany; ^5^ Institute of Clinical Chemistry and Laboratory Medicine University Medicine Greifswald Greifswald Germany; ^6^ Bioinformatics Core Weill Cornell Medicine‐Qatar, Education City Doha Qatar; ^7^ Englander Institute for Precision Medicine Weill Cornell Medicine, Belfer Research Building New York New York USA; ^8^ Interfaculty Institute for Genetics and Functional Genomics, Department of Functional Genomics University Medicine Greifswald Greifswald Germany

**Keywords:** cohort study, methylxanthine, oral care, periodontitis, theobromine

## Abstract

**Background:**

Theobromine, a methylxanthine mainly found in chocolate, has been suggested to possess various health‐promoting properties. This study aimed to investigate the long‐term effect of salivary theobromine levels on periodontitis severity using 7‐ and 10‐year follow‐up data from the prospective Studies of Health in Pomerania (SHIP‐TREND and SHIP‐START).

**Materials and Methods:**

We conducted a non‐targeted metabolomics analysis of salivary methylxanthines in 679 participants from SHIP‐TREND and 953 participants from SHIP‐START. Inverse‐probability‐of‐treatment‐weighted generalised linear models were used to assess the relationship between salivary theobromine and periodontal variables, including bleeding on probing, probing depth and clinical attachment loss.

**Results:**

Higher salivary theobromine levels were significantly associated with improved periodontal health, as evidenced by lower mean probing depth and a reduced percentage of sites with probing depth ≥ 3 mm. The results were successfully replicated in the SHIP‐START data and extended to a lower clinical attachment loss.

**Discussion:**

Our cohort studies suggest that elevated salivary theobromine levels are associated with improved periodontal parameters over 7 and 10 years. These results indicate the potential for theobromine‐containing products to support periodontal health, warranting further investigation through randomised controlled trials.

## 1 Introduction

Over the past 30 years, the prevalence of periodontitis has remained unchanged in developed countries, while dental caries has steadily declined (Kocher et al. 2024). In periodontal health, the interaction of the host response with the oral commensal biofilm maintains tissue homeostasis. When this homeostatic host‐biofilm relationship is disrupted, the biofilm burden increases, leading to gingival inflammation. Two different aetiologies of periodontitis are discussed: The first emphasises the pathogenic biofilm as the primary driver, inducing a host inflammatory response in the marginal gingiva, subsequently leading to pocket formation, periodontal ligament degradation and alveolar bone loss. This perspective implies that periodontal prevention should prioritise biofilm reduction. The second hypothesis positions the inflammatory host response as the key determinant in the onset and progression of periodontitis (Gocke et al. 2014; Sahni and Van Dyke 2023; Van Dyke and Sima 2020). According to this perspective, the therapeutic focus shifts toward preventing and resolving inflammation in the periodontal tissue (Polak et al. 2015), rather than solely targeting bacterial reduction.

Over the last few decades, there has been an intensive search for pharmacological and natural compounds as oral home care products, focusing on either antibacterial effect or modulation of the host response (Szliszka et al. 2009). Plant‐derived antimicrobial or anti‐inflammatory agents have been frequently incorporated into toothpastes or mouthwashes, demonstrating reduced plaque accumulation and gingival bleeding. However, their effect on probing depth (PD), clinical attachment level (CAL) or tooth loss remains elusive (Figuero et al. 2019, 2020; Santi et al. 2021). Methylxanthines, naturally occurring plant compounds, are commonly ingested through daily dietary sources such as tea, coffee and chocolate, which contain caffeine, theophylline and theobromine. In recent years, the health benefits associated with methylxanthine consumption, particularly caffeine, have been described in relation to cardiovascular disease, endocrine disorders and cancer (Fredholm 2011; Monteiro et al. 2016). Theobromine, another methylxanthine exhibits potent anti‐inflammatory effects and protects against oxidative stress, relevant to chronic diseases like diabetes. While caffeine also possesses antioxidant benefits, contributing to liver health and reduced cancer risk, theobromine's anti‐inflammatory properties are considered stronger. Methylxanthines exert their biological effects through three primary mechanisms: adenosine receptor antagonism, phosphodiesterase inhibition and modulation and regulation of intracellular calcium. Adenosine receptor antagonism is likely the most important physiological mechanism, as methylxanthines bind to A1 and A2A receptors, effectively blocking their inflammatory signalling pathways (Monteiro et al. 2019).

In recent analyses of the Study of Health in Pomerania (SHIP), we focused on identifying salivary metabolites associated with periodontal disease; these metabolites were derived from the host or bacteria. However, we did not explore metabolites linked to periodontal health (Andorfer et al. 2021; Liebsch et al. 2019). Since these metabolite datasets contained information on methylxanthines (caffeine, theobromine, paraxanthine and theophylline), we aimed to determine the long‐term effects of salivary methylxanthines on periodontitis severity by analysing 7‐year and 10‐year follow‐up data from the prospective Studies of Health in Pomerania (SHIP‐TREND and SHIP‐START).

## 2 Methods

### 2.1 Study Design

The SHIP project comprises two population‐based cohorts with follow‐up examinations, both conducted in north‐eastern Germany. The SHIP‐TREND‐0 baseline examination was carried out between 2008 and 2012 on 4420 participants aged 20–79 years. Saliva metabolite data were available for 935 SHIP‐TREND‐0 participants, of whom 724 had 7‐year follow‐up data (SHIP‐TREND‐1). Finally, 679 participants were included for analysis (Figure S1).

In SHIP‐START, 4307 participants were examined between 1997 and 2001. Follow‐up examinations were performed after 5 (SHIP‐START‐1), 11 (SHIP‐START‐2), 16 (SHIP‐START‐3) and 21 years (SHIP‐START‐4). This study used data from SHIP‐START‐2 (baseline), SHIP‐START‐3 and SHIP‐START‐4. For main analyses, data from 953 SHIP‐START‐2 participants with saliva metabolite and 5‐year follow‐up data (SHIP‐START‐3) were used (Figure S1).

The study protocols were approved a priori by the local Ethics Committee of the University of Greifswald and written informed consent was obtained from all the participants. Reporting followed the recommendations of the Strengthening the Reporting of Observational Studies in Epidemiology (STROBE) guidelines for observational studies that were applied.

### 2.2 Collection of Saliva and Serum Samples

In SHIP‐START‐2 and SHIP‐TREND‐0, saliva samples were taken immediately after the dental interview, before any further oral examinations were performed. The participants were asked to refrain from eating, drinking or smoking beforehand. They had to rinse their mouths with clear water three times before saliva sampling. The participants chewed on a plain cotton roll for 1 min to stimulate saliva using a hygienic collection system (Salivette; Sarstedt, Germany). The rolls of collected saliva were placed in the Salivette tube and immediately centrifuged at 1000*g* for 20 min at 4°C to remove food debris, insoluble material and cell debris. The resulting supernatant was stored at −80°C (Andorfer et al. 2021; Liebsch et al. 2019). Blood samples were collected from the cubital vein of the participants in the supine position between 6:00 am and 1:00 pm and a conventional full blood count was analysed immediately or aliquots were stored for further analyses at −80°C in the Integrated Research Biobank (LiCONiC, Mauren, Liechtenstein) of the University Medicine Greifswald.

### 2.3 Serum Metabolome in SHIP‐TREND‐0

The SHIP‐TREND‐0 samples were analysed in 2013. Non‐targeted metabolomics analysis for metabolic profiling was conducted at the Genome Analysis Centre, Helmholtz Centre Munich. Two separate ultra‐high‐performance liquid chromatography and tandem mass spectrometry (UHPLC–MS/MS) analytical methods (i.e., in positive and negative ionisation modes) were used, as previously published (Evans et al. 2009), to obtain a broad metabolite spectrum in saliva samples in a non‐targeted manner. Several preprocessing steps were performed, as described in detail in Appendix provided in the Supporting Information. After preprocessing, 284 saliva metabolites remained for statistical analysis. Of these, 105 could not be unambiguously assigned to a chemical identity and are denoted with an X followed by a unique number.

### 2.4 Serum Metabolome in SHIP‐START‐2

The SHIP‐START‐2 samples were analysed in 2018. Metabolic profiling of saliva samples was conducted by Metabolon Inc. (Hallbergmoos, Germany) using the Metabolon Discovery HD4 platform. For each sample, four different ultra‐high‐performance liquid chromatography–tandem mass spectrometry (UPLC‐MS/MS) methods were applied to allow for maximum coverage of the small molecule content. Several preprocessing steps were performed, as described in detail in the Appendix provided in the Supporting Information. Metabolon used an internal reference library with more than 3000 pure chemical compounds to identify metabolites based on retention time/index, mass to charge ratio and chromatographic data. All named compounds fulfill tier 1 or tier 2 (indicated by a star) criteria according to the metabolomics reporting standards (Sumner et al. 2007). Additional mass spectral entries have been created for structurally unnamed biochemicals, which have been identified by virtue of their recurrent nature (both chromatographic and mass spectral). After processing, 562 saliva metabolites remained for statistical analyses. Out of these, 64 metabolites could not be unambiguously assigned to a chemical identity and are designated to hereafter with an X, combined with a unique number.

Non‐parametric multiplicative Kaplan–Meier smoothing spline replacement was used to impute left‐censored values below the detection limit. Information on censoring levels, numbers of censored values and distributions before and after imputation is given in Table S1.

### 2.5 Oral Examination

The examinations were conducted in a dental chair with light and without saliva ejector or air jet. Using the half‐mouth method (SHIP‐START: alternating on the left or right side; SHIP‐TREND: left or right side, randomly selected) PD and CAL were assessed at four sites (mesiobuccal, midbuccal, distobuccal and midpalatinal/midlingual) of all teeth except the third molars using a periodontal probe (SHIP‐START: PCP‐11; SHIP‐TREND: PCP‐15; Hu‐Friedy, Chicago, USA). The presence of bleeding on probing (BOP) was recorded at the same four sites on the first incisor, canine and first molar. If a tooth was missing, the next distally located tooth was considered. The percentage of sites with bleeding on probing (%BOP) was calculated. The following oral examination variables were derived: %BOP, mean PD, the percentage of sites with PD ≥ 3 mm (%PD3 + mm), mean CAL.

### 2.6 Calibration Data

Calibrated and licensed dentists performed the dental examinations. Calibration exercises were conducted every 6–12 months with 4 to 5 persons not involved in the study. Calibration data are reported in detail in the Appendix provided in the Supporting Information.

### 2.7 Covariates

Confounders were chosen according to the modified disjunctive cause criterion (VanderWeele 2019). Sociodemographic and behavioural parameters were assessed by computer‐assisted personal interviews. Smoking status was classified as never, former and current smoker. Standardised measurements of body height and weight were made with calibrated scales, and the body mass index (BMI) was calculated as weight divided by height^2^ (kg/m^2^). Blood samples were drawn from the cubital vein in the supine position and aliquots were prepared for immediate analysis and for storage at −80°C. Serum magnesium concentrations were measured using an automated biochemical analyser (Cobas Integra 800, Roche Diagnostics, Mannheim, Germany). HbA1c concentrations were determined by high‐performance liquid chromatography (Bio‐Rad Diamant, Munich, Germany). Diabetes mellitus was defined as having known diabetes or glycated haemoglobin level of (HbA1c) ≥ 6.5% or non‐fasting serum glucose ≥ 11.1 mmol/L; known diabetes mellitus was defined as physician's diagnosis or antidiabetic medication intake (Anatomic Therapeutic Chemical classification system; code A10). Based on toothbrushing frequency, the participants were categorised as irregular (< 2 times/day) or regular tooth brushers (≥ 2 times/day). Use of interdental aids was defined as the self‐reported daily use of wooden sticks (toothpicks and wooden tooth sticks), dental floss or interdental brushes (yes/no), tooth brush usage was defined either as manual (MTB) or powered tooth brush (PTB) usage, and the participants who used both toothbrushes were classified as PTB users. Dental visit within the last year was defined as yes/no. The follow‐up time of the examinations was given in exact years.

### 2.8 Statistical Analyses

Means with standard deviations (SDs) and medians with 25% and 75% quantiles were reported for continuous variables. Absolute and relative frequency distributions were computed for categorical variables.

The associations of saliva metabolites (exposure) with dental variables (outcome) were modelled using generalised linear models with gamma distribution and log link (mean PD, mean CAL; reporting exponentiated beta coefficients with 95% confidence intervals and interpreted as a percent change of the outcome; Manning et al. 2005), fractional response logit models (%BOP and %PD3+ mm; reporting exponentiated beta coefficients with 95% confidence intervals and interpreted as a percent change of the outcome; Papke and Wooldridge 1996).

Second, we used SHIP‐START‐2, SHIP‐START‐3 and SHIP‐START‐4 data to run mixed models (random‐effects terms included intercept and time). Generalised linear mixed models with gamma distribution and log link (mean PD, mean CAL; reporting exponentiated beta coefficients with 95% confidence intervals) and linear mixed models (%BOP, %PD3+ mm; reporting beta coefficients with 95% confidence intervals) were evaluated.

We applied a covariate balancing propensity score to estimate a generalised propensity score (Fong et al. 2018) and inverse probability treatment weighting (IPTW) to adjust for confounding. Confounders included age (cont.), sex, school education, smoking, known or diagnosed diabetes mellitus, body mass index (cont.), tooth brushing frequency, use of interdental cleaning aids and PTB use. Non‐linear associations of continuous variables were modelled using restricted cubic splines with three knots. Weights were stabilised and trimmed at 1% and 99%. We assessed balance using weighted correlations between each covariate and the continuous exposure, where standardised mean differences < 0.10 indicate good balance. A robust sandwich variance estimator was used to calculate the standard errors (Chesnaye et al. 2022).

As we aimed to estimate the total effects of saliva metabolites on periodontal variables, we did not adjust models for baseline periodontal status, as it was assumed to be a mediator of the effect of metabolite levels on follow‐up periodontal status (Tennant et al. 2022), assuming long‐term a priori effects of saliva metabolites on baseline periodontal/dental status. Furthermore, as reverse causality (VanderWeele et al. 2016) was not an issue in this specific sample (all metabolites are only ingested with food), we did not adjust for baseline periodontal status. We estimated a generalised propensity score using the covariate balancing propensity score.

We performed several sensitivity analyses. Firstly, we repeated all analyses using regression adjustment, instead of IPTW, to adjust for confounding. Secondly, models were additionally adjusted for baseline levels of serum magnesium, because dark chocolate is not only an excellent source of theobromine (Cinquanta et al. 2016), but also of magnesium and an adequate magnesium serum level may prevent progression of periodontitis and tooth loss (Meisel et al. 2016).

A two‐sided *p* < 0.05 was considered statistically significant. All analyses were performed using Stata/SE Version 17.0 (StataCorp 2021) and R 4.1.2 (R Core Team Vienna 2021).

## 3 Results

### 3.1 Baseline Characteristics

A detailed description of the SHIP‐TREND‐0 and SHIP‐START‐2 study populations is provided in Table 1. In brief, SHIP‐TREND‐0 participants had a mean age of 49.2 years, were equally between women and men, and approximately 42.3% were never smokers. The baseline values of mean and mean CAL were 2.48 ± 0.58 mm and 2.16 ± 1.32 mm, respectively, and the follow‐up values were 2.34 ± 0.54 mm and 2.16 ± 1.17 mm, respectively. The mean age of the SHIP‐START‐2 participants was 52.5 years. The baseline values for mean PD and mean CAL were 2.55 ± 0.48 mm and 2.56 ± 1.35 mm, respectively, and the follow‐up values were 2.42 ± 0.51 mm and 2.42 ± 1.33 mm, respectively.

**TABLE 1:** Baseline characteristics of SHIP‐TREND‐0 and SHIP‐START‐2 study populations. For oral variables, 7‐year and 5‐year follow‐up levels are additionally provided.

	N	SHIP‐TREND‐0	N	SHIP‐START‐2
Age, years	679	49.2 ± 12.8 49 (40; 59)	953	52.5 ± 11.9 52 (43; 61)
Male sex	679	296 (43.6%)	953	482 (50.6%)
School education	679		953	
< 10 years		56 (8.3%)		147 (15.4%)
10 years		386 (56.8%)		571 (59.9%)
> 10 years		237 (34.9%)		235 (24.7%)
Smoking status	679		953	
Never smoker		287 (42.3%)		366 (38.4%)
Former smoker		265 (39.0%)		408 (42.8%)
Current smoker		127 (18.7%)		179 (18.8%)
Body mass index, kg/m^2^	679	27.1 ± 4.3 26.7 (24.1; 29.6)	953	27.7 ± 4.6 26.9 (24.4; 30.4)
Known or diagnosed diabetes mellitus, yes	679	19 (2.8%)	953	77 (8.1%)
Tooth brushing frequency	679		953	
Irregular brushers (< 2 times daily)		65 (9.6%)		122 (12.8%)
Regular brushers (≥ 2 times daily)		614 (90.4%)		831 (87.2%)
Use of interdental cleaning aids, yes	679	209 (30.8%)	953	303 (31.8%)
Use of powered toothbrushes, yes	679	213 (31.4%)	953	317 (33.3%)
Dental visit within the last year, yes	679	636 (93.7%)	953	883 (92.7%)
Serum magnesium, mmol/L	679	0.89 ± 0.08 0.89 (0.84; 0.94)	953	0.84 ± 0.09 0.84 (0.78; 0.91)
Baseline saliva metabolite status				
Caffeine	679	1.84 ± 2.26 1.12 (0.47; 2.28)	953	1.23 ± 1.07 0.99 (0.49; 1.66)
Paraxanthine	679	1.40 ± 1.17 1.15 (0.65; 1.76)	953	1.09 ± 0.72 0.99 (0.57; 1.42)
Theobromine	679	1.57 ± 1.39 1.15 (0.71; 1.98)	953	1.26 ± 0.98 1.00 (0.57; 1.67)
Theophylline	679	1.19 ± 1.41 0.95 (0.41; 1.51)	953	1.11 ± 0.78 1.00 (0.58; 1.46)
Baseline oral status				
Percentage of sites with bleeding on probing, %	676	24.5 ± 23.3 20 (4.2; 37.5)	953	22.3 ± 20.5 16.7 (6.3; 33.3)
Mean PD, mm	675	2.48 ± 0.58 2.37 (2.11; 2.65)	953	2.55 ± 0.48 2.46 (2.23; 2.77)
Percentage of sites with PD ≥ 3 mm, %	675	39.5 ± 20.6 37.5 (25; 50)	953	48.6 ± 20.0 47.9 (34.1; 61.5)
Mean CAL, mm	643	2.16 ± 1.32 1.92 (1.18; 2.88)	953	2.56 ± 1.35 2.39 (1.57; 3.26)
Follow‐up oral status				
Percentage of sites with bleeding on probing, %	679	17.7 ± 18.8 12.5 (4.2; 25)	953	22.3 ± 19.3 16.7 (8.3; 33.3)
Mean PD, mm	678	2.34 ± 0.54 2.23 (2.04; 2.5)	953	2.42 ± 0.51 2.33 (2.11; 2.60)
Percentage of sites with PD ≥ 3 mm, %	678	31.7 ± 19.2 28.6 (16.7; 43.8)	953	42.8 ± 18.6 41.7 (29.2; 55.4)
Mean CAL, mm	645	2.16 ± 1.17 1.83 (1.40; 2.63)	953	2.42 ± 1.33 2.07 (1.54; 2.95)

*Note*: Data are presented as mean ± standard deviation and median (25%; 75% quantiles) or number (percentage). The unit for metabolites is normalised dimensionless intensities.

Abbreviations: CAL, clinical attachment level; DFS, number of decayed or filled surfaces; *N*, number; PD, probing depth.

### 3.2 Association of Salivary Metabolites on Periodontal Status at Follow‐Up

**FIGURE 1:**
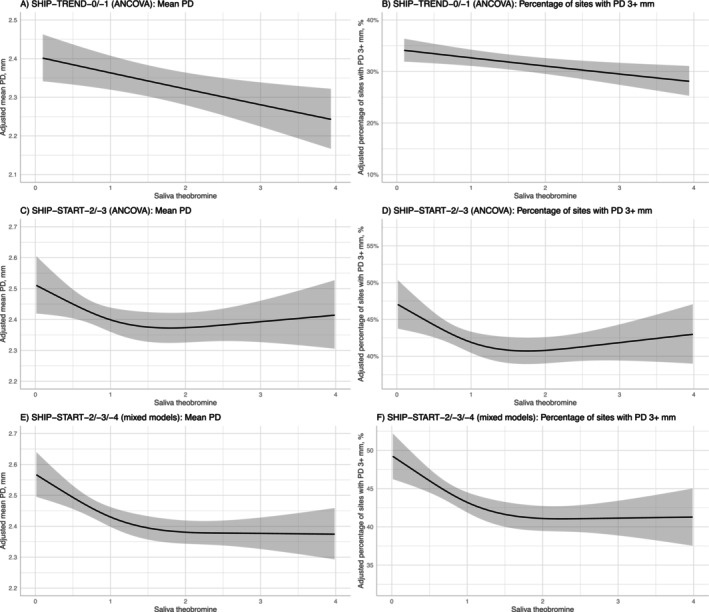
Predicted values of mean probing depth (left column) and the percentage of sites with probing depths 3+ mm (right column) for varying levels of saliva theobromine (*x* axis; presented for saliva theobromine levels up to the 95th percentile) retrieved from either inverse probability treatment weighted analysis of covariance (ANCOVA) models using SHIP‐TREND (A, B; for models see Table 2) or SHIP‐START (C, D; for models see Table S3) data, or inverse probability treatment weighted mixed models using SHIP‐START data (E, F; for models, see Table S5). PD, probing depth.

First, we evaluated the associations of baseline levels of salivary metabolites with periodontal status at follow‐up using SHIP‐TREND data (Table 2 and Figure 1A,B). Higher levels of theobromine were associated with lower mean PD (exp(beta) = 0.982; 95% confidence interval [CI]: 0.972–0.993) and fewer pockets with PD3+ mm (exp(beta) = 0.929; 95% CI: 0.885–0.976). Results were consistent for regression‐adjusted models (Table S2). The inclusion of magnesium did not change any of the coefficients (Table S3).

**TABLE 2:** Results from inverse probability treatment weighted models using baseline and 7‐year follow‐up data of SHIP‐TREND.

	Percentage of sites with bleeding on probing (*N* = 679)	Mean PD (*N* = 678)	Percentage of sites with PD ≥ 3 mm (*N* = 678)	Mean CAL (*N* = 645)
	Exp(beta) with 95% CI	Exp(beta) with 95% CI	Exp(beta) with 95% CI	Exp(beta) with 95% CI
Caffeine	1.024 (0.978; 1.071)	0.997 (0.988; 1.006)	0.992 (0.956; 1.030)	1.020 (0.992; 1.048)
Paraxanthine	1.032 (0.953; 1.118)	0.992 (0.978; 1.007)	0.980 (0.922; 1.041)	1.025 (0.969; 1.084)
Theobromine	0.981 (0.912; 1.056)	**0.982 (0.972; 0.993)**	**0.929 (0.885; 0.976)**	0.993 (0.954; 1.033)
Theophylline	1.009 (0.906; 1.124)	0.992 (0.973; 1.011)	0.971 (0.895; 1.054)	1.033 (0.961; 1.112)

*Note*: Generalised linear models with gamma distribution and log link for mean PD and mean CAL (reporting exponentiated beta coefficients with 95% confidence intervals), fractional response models for the percentage of sites with bleeding on probing and the percentage of sites with PD ≥ 3 mm (reporting exponentiated beta coefficients with 95% confidence intervals) and negative binomial regression models for incident tooth loss (reporting incidence rate ratios with 95% confidence intervals). Models were adjusted for age (cont.), sex, school education, smoking, known or diagnosed diabetes mellitus, body mass index (cont.), tooth brushing frequency, use of interdental cleaning aids and PTB use. The unit for metabolites is normalised dimensionless intensities. Bold means *p* value < 0.05.

Abbreviations: CAL, clinical attachment level; CI, confidence interval; IRR, incidence rate ratio; *N*, number; PD, probing depth; RCS, restricted cubic spline term.

Second, we tested whether the associations of theobromine on PD variables replicated in SHIP‐START (Table S4). Higher theobromine levels were associated with lower levels of mean PD and %PD3+ mm (Figure 1C,D), although associations were attenuated at theobromine levels of ≥ 2. Furthermore, we found the association of paraxanthine on %BOP in both IPTW (exp(beta) = 0.904; 95% CI: 0.819; 0.998). The inclusion of magnesium did not change any of the coefficients (Table S5).

**FIGURE 2:**
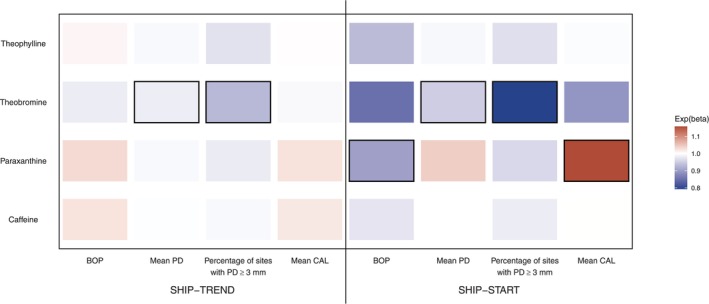
This heat plot illustrates the associations of four methylxanthines (theophylline, theobromine, paraxanthine and caffeine) with four periodontal parameters (bleeding on probing [BOP], mean probing depth [PD], percentage of sites with PD ≥ 3 mm and mean clinical attachment level [CAL]) in two cohorts: SHIP‐TREND (7‐year follow‐up) and SHIP‐START (10‐year follow‐up). The colour gradient represents the effect size (exp(beta)), with blue indicating a negative association and red indicating a positive association. The boxed cells represent statistically significant associations (*p* value < 0.05).

Third, we tested whether the associations of theobromine on PD and CAL variables replicated in SHIP‐START using data from three waves, that is, SHIP‐START‐2 to −4 (Figures 1E,F and 2, Table S6). Associations of theobromine on mean PD and %PD3+ mm were confirmed, again with attenuated associations at theobromine levels of ≥ 2 (Figure 1E,F). In contrast to shorter follow‐up periods, higher theobromine levels were associated with lower CAL values (restricted cubic spline term 1: exp(beta) = 0.942 [0.911; 0.975]). We also found significant associations of paraxanthine on mean PD, %PD3+ mm and mean CAL. The inclusion of magnesium did not change any of the coefficients (Figure 2, Table S6).

## 4 Discussion

This is the first longitudinal cohort study to show that higher salivary levels of theobromine are associated with improved periodontal health at follow‐up levels, as measured by mean PD and %PD3+ mm. The results were successfully replicated using data from an independent cohort (SHIP‐START). The strength of effect of theobromine on mean PD and %PD3+ mm increased with longer follow‐up time (Figure 2). Most notably, we observed a significant improvement in the CAL, which reflects the cumulative history of periodontitis and is the more critical parameter in the long term than PD: This is because tooth loss due to periodontitis is more strongly associated with CAL than with PD. Although the associations of other methylxanthines were not statistically significant, higher salivary levels of caffeine, paraxanthine and theophylline tended to be associated with lower mean PD and %PD3+ mm.

The findings of this study are consistent with those of a recent analysis of the National Health and Nutrition Examination Survey (NHANES), which demonstrated that theobromine derived from a two‐day total nutrient intake was inversely correlated with mean periodontal pocket depth, mean clinical attachment loss and the percentage of sites with PD ≥ 4 mm and was positively associated with the number of teeth (Zhou et al. 2025).

To understand whether the observed protective effect of theobromine could be clinically relevant, we have to enter uncertain territory. An increase of 1 unit of theobromine spans the range of the 25th to 75th quartile and is associated with a change of 2%–3% in mean PD, corresponding to 0.09 mm (Table 2) or a difference of 0.1 mm between 0 and 1 unit of salivary theobromine (Figure 1E). The critical question is whether a mean PD reduction of 0.1 mm in over 7 years constitutes a clinically significant improvement. In SHIP‐START, participants who used PTB had significantly less PD progression of 0.09 mm than MTB brushers after 11 years of follow‐up (Pitchika et al. 2019). An Oaxaca decomposition analysis of two national cross‐sectional studies 7 years apart showed that out of a predicted observed PD improvement of 0.12 mm in adults (0.17 mm in seniors), a reduction of 0.05 mm in adults (0.09 mm in seniors) was attributable to the use of interdental cleaning aids (Pitchika et al. 2021). Based on these findings, we can conclude that a 1 unit increase in theobromine corresponds to a magnitude of improvement comparable to that achieved with an electric toothbrush or interdental cleaning aids, both of which are recognised by the periodontal community as essential tools for periodontal health.

In the human body, cocoa consumption represents the primary natural source of theobromine, accounting for 98% of its intake (Health 1991). Cocoa contains more theobromine than caffeine, and theobromine is likely responsible for many of the health benefits attributed to coffee or chocolate (Martinez‐Pinilla et al. 2015; Neufingerl et al. 2013). Theobromine is found in the highest concentrations in cocoa products such as chocolate, candy bars, cakes and puddings, and in smaller concentrations in coffee and tea. Many studies have identified theobromine as a potential biomarker of cocoa consumption (Goerdten et al. 2024). Although theobromine has a half‐life of around 6–10 h (Rodopoulos et al. 1996), its presence in saliva may indicate habitual dietary patterns. A recent NHANES publication associated dietary theobromine intake with periodontitis variables and producing results comparable to ours (Zhou et al. 2025). According to consumer surveys, 75% of Germans eat chocolate at least once a week, with over 10% daily (Mintel 2024) and 92% regularly drink coffee, with 68% doing so daily (Aral 2025). Therefore, a statistically protective association in saliva could represent long‐term biological effects.

The pharmacological mechanisms of methylxanthines involve competitive antagonism of adenosine receptors, inhibition of phosphodiesterase and regulation of intracellular calcium levels (Martinez‐Pinilla et al. 2015; Monteiro et al. 2016). Specially, stimulation of adenosine receptors A2A and A2B increases extracellular AMP (Monteiro et al. 2016), which exacerbates inflammation (Pasquini et al. 2021) and promotes leukocyte recruitment in periodontitis (Binderman et al. 2017). The primary pharmacological effect of methylxanthines is probably due to the antagonistic binding to A1 and A2A receptors (Fredholm 2011). For instance, in a rat model of experimental periodontitis, blocking the A2A receptor resulted in reduced release of pro‐inflammatory mediators such as TNF‐α and IL‐6, as well as attenuation of excessive periodontal tissue destruction (Bitto et al. 2013). Additionally, chemically modified synthetic xanthines have shown anti‐inflammatory properties, including suppressed RANKL‐induced osteoclastogenesis, and stimulated osteoblast differentiation (Huang et al. 2025, 2021; Kuo et al. 2022). The results presented in these experimental animal models support our conclusion that theobromine may indeed support periodontal health.

A key question is why only theobromine exhibited a significant effect on periodontal health, while other methylxanthines did not. Despite similar chemical structures, one possible explanation for the different pharmacological effects lies in the slower metabolic rate of theobromine (Martinez‐Pinilla et al. 2015) and its longer half‐life (7.2 h) compared to caffeine (4.2 h) (Monteiro et al. 2019). Another explanation is that theobromine more easily crosses biological compartments, potentially allowing it to accumulate at higher concentrations in the gingival tissues (Martinez‐Pinilla et al. 2015). Furthermore, caffeine is rapidly metabolised to paraxanthine and theophylline, which exhibit different adenosine receptor affinities than theobromine, whereas theobromine is not metabolised to these metabolites (Fredholm 2011).

Based on these findings, it may be worthwhile for the oral care industry to explore the development of oral care products containing theobromine as a means to support periodontal health. Toothpastes and mouthwashes are regulated as cosmetic rather than pharmacological products, and methylxanthines are already used as ingredients in cosmetic products, where they are considered safe (Agency 2020; Cherian et al. 2018). Therefore, the oral care industry could timely study the effect of theobromine‐containing mouthwashes and toothpastes and at least clarify three different aspects: (i) whether the observed effect of theobromine is induced by bathing the periodontal tissues with theobromine from saliva or as a transudate passing through the periodontal tissues from the blood circulation, because a local effect could only be expected from bathing with theobromine from the saliva. On the other hand, if the effect comes mainly from the transudate, it would be a systemic effect, which is not consistent with the use of an oral care product, (ii) the dose required to achieve periodontal health benefits and (iii) whether a sufficiently high concentration can be achieved with rinsing or tooth brushing. Our findings call for the oral care industry to investigate theobromine‐containing products for primary prevention.

A strength of our study is the prospective longitudinal study design and the long median follow‐up of 7 years in SHIP‐TREND and 5 (11) years in SHIP‐START. We studied several methylxanthine compounds and several oral health outcomes. As each compound was measured quantitatively, we were able to demonstrate that only theobromine but not the other methylxanthines had an effect on an attenuated progression of periodontitis. We carefully selected regression models, including comprehensive adjustment for various confounders, to minimise the chance of residual confounding. We did not adjust models for baseline periodontal status, as it was assumed to be a mediator of the effect of metabolite levels on follow‐up periodontal/caries status (Tennant et al. 2022), assuming long‐term a priori effects of salivary metabolites on baseline periodontal status. Furthermore, we did not adjust for baseline periodontal as reverse causality (VanderWeele et al. 2016) was not an issue in this specific sample (all metabolites are only ingested with food).

This study has some limitations. The main source of theobromine is chocolate. The observed association between theobromine and periodontitis is susceptible to confounding, because higher levels of theobromine in chocolate are associated with higher levels of magnesium and bioactive compounds such as antioxidants, polyphenols and flavonoids. These compounds contribute to overall health through mechanisms such as antioxidant activity and reduction of inflammation. One randomised controlled trial (RCT) concluded that theobromine is the main component responsible for the HDL cholesterol‐raising effect (Neufingerl et al. 2013). However, a large RCT investigating the potential of flavanol‐enriched cocoa bars failed to show a statistically significant reduction in cardiovascular disease (CVD) incidence in the test group (Sesso et al. 2022). In addition, we performed a sensitivity analysis including magnesium, because magnesium is protective against periodontitis (Meisel et al. 2005). The inclusion of magnesium did not change the influence of theobromine on periodontitis. In addition, only baseline methylxanthine levels (exposure) were included in the regression models, thus ignoring potential shifts in methylxanthine levels between baseline and follow‐up. Finally, measures of periodontitis were only assessed on a half‐mouth basis at four sites per tooth. Therefore, the outcome variables analysed may not reflect the true situation and mays underestimate disease severity. However, for measurements such as mean PD/CAL, the degree of bias associated with half‐mouth recordings is small (Kingman et al. 2008). In general, there is a shift in effect estimates toward the null effect (Akinkugbe et al. 2015).

## 5 Conclusions

Increased salivary levels of theobromine have been associated with reduced periodontal burden after 7 or 10 years in observational studies. Based on these findings, the oral care industry could investigate if theobromine‐containing toothpastes or mouthwashes support periodontal health and investigate their effects on periodontal status in randomised controlled trials.

## Author Contributions

T.K., B.H. and K.S. substantially contributed to the conception or design of the work. T.K., BH, S.‐E.B., N.F. and M.N. contributed to the analysis, or interpretation of data. T.K., B.H., P.M. and S.‐E.B. drafted the work. P.M., U.V., H.V. and M.N. revised the work critically for important intellectual content. All authors approved the final version of the manuscript and are accountable for all aspects of the work.

## Funding

SHIP is part of the Community Medicine Research Network of the University Medicine Greifswald, which is supported by the German Federal State of Mecklenburg‐West Pomerania. The Suhre lab is supported by the Biomedical Research Program at Weill Cornell Medicine in Qatar, a programme funded by the Qatar Foundation, and by Qatar National Research Fund (QNRF) grants NPRP11C‐0115‐180010 and ARG01‐0420‐230007.

## Ethics Statement

SHIP‐TREND was positively evaluated by the ethics committee of the University of Greifswald (SHIP‐START‐2: BB 39/08; SHIP‐START‐3: BB 122/13, SHIP‐START‐4: BB 055/19, SHIP‐TREND‐0: BB 39/08a; SHIP‐TREND‐1: BB 174/15). All the participants were informed about the study protocol and signed the informed consent and the privacy statement.

## Consent

The authors have nothing to report.

## Conflicts of Interest

The authors declare no conflicts of interest.

## Supporting information


**Data S1:** jcpe70072‐sup‐0001‐Supinfo.docx.

## Data Availability

The data that support the findings of this study are available from Forschungsverbund Community Medicine. Restrictions apply to the availability of these data, which were used under licence for this study. Data are available from https://transfer.ship‐med.uni‐greifswald.de/FAIRequest/data‐use‐intro with the permission of Forschungsverbund Community Medicine.
