# Supplementary material for: Elevated Salivary Theobromine and Long‐Term Improvement of Periodontal Health in Two Cohort Studies
Source: J Clin Periodontol. 2025 Dec 12;53(3):445–54. doi: 10.1111/jcpe.70072 (PMC12890454; doi:10.1111/jcpe.70072)
Supplement: Supplementary file 1 — Data S1: jcpe70072‐sup‐0001‐Supinfo.docx. [file JCPE-53-445-s001.docx]

# Online Only Appendix

# Material and Methods

## Calibration data

Calibrated and licensed dentists performed the dental examinations. Calibration exercises were conducted every 6-12 months with 4 to 5 persons not involved in the study.

In SHIP-START-2, dental examinations were performed by six examiners. In the calibration exercises, the dentists achieved intra-class correlations of 0.76-0.88 per examiner and an inter-class correlation of 0.74 for CAL. For PD, the intra-class correlation was 0.70-0.78 per examiner and the inter-class correlation was 0.70. For assessment of the tooth status, Cohen’s kappa reliability coefficients were 0.93-0.99 (intra-examiner) and 0.94-0.98 (pairwise inter-examiner).

In SHIP-START-3, dental examinations were performed by six examiners. In the calibration exercises, the dentists achieved intra-class correlations of 0.92-0.97 per examiner and an inter-class correlation of 0.98 for CAL. For PD, the intra-class correlation was 0.69-0.92 per examiner and inter-class correlation was 0.91. For assessment of the tooth status, Cohen’s kappa reliability coefficients were 0.93-1.00 (intra-examiner) and 0.91-0.96 (pairwise inter-examiner).

In SHIP-START-4, dental examinations were performed by five examiners. In the calibration exercises, the dentists achieved intra-class correlations of 0.90-0.96 per examiner and pairwise inter-class correlations between 0.86 and 0.94 for CAL. For PD, the intra-class correlation was 0.77-0.91 per examiner and pairwise inter-class correlations ranged between 0.63 and 0.85. For the assessment of the tooth status, Cohen’s kappa reliability coefficients were 0.97-1.00 (intra-examiner) and 0.91-0.96 (pairwise inter-examiner).

In SHIP-TREND-0, dental examinations were conducted by six calibrated examiners. Intra-rater correlations for CAL measurements ranged between 0.67 and 0.89 and inter-rater correlation was 0.70. For PD measurements, the examiners yielded intra-rater correlations between 0.68 and 0.88 and an inter-rater correlation of 0.72. For assessment of the tooth status, Cohen’s kappa reliability coefficients were 0.93-0.99 (intra-examiner) and 0.94-0.98 (pairwise inter-examiner).

In SHIP-TREND-1, dental examinations were performed by six calibrated examiners. Intra-examiner correlation for CAL values varied from 0.90-0.96. Pairwise inter-examiner correlations ranged between 0.86-0.94. For PD values intra-examiner correlations ranged from 0.77 to 0.91. Pairwise inter-examiner correlations ranged from 0.63-0.85. Cohen’s kappa reliability coefficients for assessment of the tooth status were 0.97-1.00 (intra-examiner) and 0.91-0.96 (pairwise inter-examiner).

## Metabolomic measurements: preprocessing steps

On the day of extraction, samples were thawed on ice. A 100μL of the sample were pipetted into a 2mL 96-well plate. In addition to study samples, a human pooled reference sample (Seralab, West Sussex, United Kingdom) and another pooled reference matrix of each sample set (Seralab, West Sussex, United Kingdom) were extracted and placed in 1 and 6 wells, respectively, of the 96-well plate. These samples served as technical replicates throughout the data set to assess process variability. Beside those samples, 100μL of water was extracted as samples and placed in 6 wells of the 96-well plate to serve as process blanks. Protein was precipitated and the metabolites were extracted with 475μL methanol, containing four recovery standards to monitor the extraction efficiency. After centrifugation, the supernatant was split into 4 aliquots of 100μL each onto two 96-well microplates. The first 2 aliquots were used for LC-MS/MS analysis in positive and negative electrospray ionization mode. Two further aliquots were kept as a reserve. The extracts were dried on a TurboVap 96 (Zymark, Sotax, Lörrach, Germany). Prior to LC-MS/MS in positive ion mode, the samples were reconstituted with 0.1% formic acid. Whereas samples analyzed in negative ion mode were reconstituted with 6.5mM ammonium bicarbonate, pH 8.0. Reconstitution solvents for both ionization modes contained internal standards that allowed monitoring of instrument performance and also served as retention reference markers. To minimize human error, liquid handling was performed on a Hamilton Microlab STAR robot (Hamilton Bonaduz AG, Bonaduz, Switzerland). LC-MS/MS analysis was performed on a linear ion trap LTQ XL mass spectrometer (Thermo Fisher Scientific GmbH, Dreieich, Germany) coupled with a Waters Acquity UPLC system (Waters GmbH, Eschborn, Germany). Two separate columns (2.1 x 100 mm Waters BEH C18, 1.7 μm particle-size) were used either for acidic (solvent A: 0.1% formic acid in water, solvent B: 0.1% formic acid in methanol) and or for basic (A: 6.5mM ammonium bicarbonate, pH 8.0, B: 6.5mM ammonium bicarbonate in 95% methanol) mobile phase conditions, optimized for positive and negative electrospray ionization, respectively. After injection of the sample extracts, the columns were developed in a gradient of 99.5% A to 98% B over an 11 min run time at 350 μLl/min flow rate. The eluent flow was directly run through the ESI source of the LTQ XL mass spectrometer. The mass spectrometer analysis alternated between MS and data-dependent MS/MS scans using dynamic exclusion and the scan range was from 80-1000 m/z. Metabolites were identified by Metabolon, Inc., from the LC-MS/MS data by automated multiparametric comparison with a proprietary library, containing retention times, m/z ratios, and related adduct/fragment spectra (Lawton et al., 2008). Identification criteria for the detected metabolites are described in (Evans et al., 2009).

## Metabolomic measurements: Quality control and normalization of metabolite levels

To correct for daily variations of platform performance, the raw ion count of each metabolite was rescaled by the respective median value of the run day. Valid estimation of the median was ensured by keeping only metabolites with at least three measured values on more than the half of the run days. We chose probabilistic quotient normalization (PQN) (Dieterle et al., 2006) to account for diurnal variation of saliva samples. For this purpose, we calculated a mean-pseudo-spectrum depending on metabolites with measurements for all participants (37 saliva metabolites). Subsequently, we calculated a dilution factor as the median quotient between the reference spectrum and each sample. Afterwards all metabolite levels were log2-transformed. We performed multivariate outlier detection using an algorithm proposed by (Filzmoser et al., 2008) as implemented in the pcout function within the R package mvoutlier. The algorithm provides an outlier score for each sample based on a weighted combination of location and scatter estimations using principle component analysis and the Mahalanobis distance on a robustly scaled data matrix. The default parameters were used for the identification process, except the critical value for the location outliers was set to 4, as it corresponds to a 4 SD exclusion criterion. The minimum score was used as cut-off for outlier identification.

## References

Dieterle, F., Ross, A., Schlotterbeck, G., & Senn, H. (2006). Probabilistic quotient normalization as robust method to account for dilution of complex biological mixtures. Application in 1H NMR metabonomics. *Anal Chem*, *78*(13), 4281-4290. <https://doi.org/10.1021/ac051632c>

Evans, A. M., DeHaven, C. D., Barrett, T., Mitchell, M., & Milgram, E. (2009). Integrated, nontargeted ultrahigh performance liquid chromatography/electrospray ionization tandem mass spectrometry platform for the identification and relative quantification of the small-molecule complement of biological systems. *Anal Chem*, *81*(16), 6656-6667. <https://doi.org/10.1021/ac901536h>

Filzmoser, P., Maronna, R., & Werner, M. (2008). Outlier identification in high dimensions. *Computational Statistics & Data Analysis*, *52*(3), 1694-1711.

Lawton, K. A., Berger, A., Mitchell, M., Milgram, K. E., Evans, A. M., Guo, L., Hanson, R. W., Kalhan, S. C., Ryals, J. A., & Milburn, M. V. (2008). Analysis of the adult human plasma metabolome. *Pharmacogenomics*, *9*(4), 383-397. <https://doi.org/10.2217/14622416.9.4.383>

Appendix Table 1. Non-parametric multiplicative Kaplan-Meier smoothing spline imputation of left-censored values below the detection limit.

|  | Non-imputed censored data | | | Imputed data |
| --- | --- | --- | --- | --- |
|  | Mean ± standard deviation | Censoring level | Number of censored values | Mean ± standard deviation |
| SHIP-TREND-0 (N=935) | | | | |
| Caffeine | 1.901186±2.271959 | 0.0788 | 35 | 1.832969±2.255695 |
| Paraxanthine | 1.436331±1.147354 | 0.0820 | 21 | 1.405913±1.152015 |
| Theobromine | 1.554392±1.340512 | 0.0994 | 6 | 1.545055±1.341246 |
| Theophylline | 1.448303±1.614882 | 0.2657 | 215 | 1.176363±1.501817 |
| SHIP-START-2 (N=999) | | | | |
| Caffeine | 1.255479±1.0691549 | 0.0048 | 17 | 1.234196±1.072293 |
| Paraxanthine | 1.104016±0.7164627 | 0.0326 | 21 | 1.081493±0.725372 |
| Theobromine | 1.249428±0.9704178 | 0.0138 | 1 | 1.248191±0.970719 |
| Theophylline | 1.128612±0.7765654 | 0.0353 | 15 | 1.112196±0.7821036 |

Appendix Table 2. Results from regression-adjusted models using baseline and 7-year follow-up data of SHIP-TREND.

|  | Percentage of sites with bleeding on probing  (N=679) | Mean PD  (N=678) | Percentage of sites  with PD ≥3 mm  (N=678) | Mean CAL  (N=645) |
| --- | --- | --- | --- | --- |
|  | Exp(beta) with 95% CI | Exp(beta) with 95% CI | Exp(beta) with 95% CI | Exp(beta) with 95% CI |
| Caffeine | 1.027 (0.983; 1.072)** | 1.063 (0.999; 1.130)* | 0.988 (0.956; 1.022)* | 1.014 (0.994; 1.034) |
| Paraxanthine | 1.023 (0.954; 1.097)** | 0.992 (0.979; 1.004)* | 0.970 (0.916; 1.027)* | 1.015 (0.976; 1.055) |
| Theobromine | 0.977 (0.915; 1.043)** | **0.983 (0.974; 0.993)*** | **0.924 (0.882; 0.968)*** | 0.988 (0.957; 1.020) |
| Theophylline | 1.062 (0.955; 1.182)** | 0.999 (0.979; 1.019)* | 0.982 (0.906; 1.065)* | 1.025 (0.972; 1.080) |

Models: Generalized linear models with gamma distribution and log link for mean PD and mean CAL (reporting exponentiated beta coefficients with 95% confidence intervals), fractional response models for the percentage of sites with bleeding on probing and the percentage of sites with PD ≥3 mm (reporting exponentiated beta coefficients with 95% confidence intervals), and negative binomial regression models for incident tooth loss (reporting Incidence Rate Ratios with 95% confidence intervals). Models were adjusted for age (cont.), sex, school education, smoking, known or diagnosed diabetes mellitus, body mass index (cont.), tooth brushing frequency, use of interdental cleaning aids, and use of powered tooth brushes. The unit for metabolites is normalised dimensionless intensities. Abbreviations: CAL, clinical attachment level; CI, confidence interval; IRR, Incidence Rate Ratio; N, number; PD, probing depth; RCS: restricted cubic spline term. * body mass index modelled as cubic splines with three knots; ** age modelled as cubic splines with three knots.

Appendix Table 3. Results from inverse probability treatment weighted models and from regression-adjusted models using baseline and 7-year follow-up data of SHIP-TREND. With additional adjustment for baseline levels of serum magnesium.

|  | Percentage of sites with bleeding on probing  (N=679) | Mean PD  (N=678) | Percentage of sites  with PD ≥3 mm  (N=678) | Mean CAL  (N=645) |
| --- | --- | --- | --- | --- |
| **Inverse probability treatment weighted models** | | | | |
|  | Exp(beta) with 95% CI | Exp(beta) with 95% CI | Exp(beta) with 95% CI | Exp(beta) with 95% CI |
| Caffeine | 1.021 (0.976; 1.068) | 0.996 (0.987; 1.006) | 0.991 (0.956; 1.029) | 1.017 (0.990; 1.045) |
| Paraxanthine | 1.033 (0.954; 1.120) | 0.992 (0.978; 1.007) | 0.979 (0.922; 1.040) | 1.026 (0.970; 1.084) |
| Theobromine | 0.979 (0.909; 1.055) | **0.982 (0.972; 0.992)** | **0.928 (0.885; 0.974)** | 0.993 (0.952; 1.036) |
| Theophylline | 1.009 (0.906; 1.125) | 0.992 (0.973; 1.011) | 0.971 (0.895; 1.054) | 1.033 (0.960; 1.112) |
| **Regression-adjusted models** | | | | |
|  | Exp(beta) with 95% CI | Exp(beta) with 95% CI | Exp(beta) with 95% CI | Exp(beta) with 95% CI |
| Caffeine | 0.026 (0.983; 1.072)** | **RSC1: 0.968 (0.938; 0.999)**  RCS2: 1.059 (0.997; 1.126)* | 0.988 (0.956; 1.022)* | 1.013 (0.994; 1.034) |
| Paraxanthine | 1.023 (0.955; 1.097)** | 0.992 (0.979; 1.004)* | 0.970 (0.916; 1.027)* | 1.015 (0.977; 1.056) |
| Theobromine | 0.976 (0.915; 1.042)** | **0.983 (0.974; 0.992)*** | **0.924 (0.882; 0.968)*** | 0.987 (0.956; 1.020) |
| Theophylline | 1.062 (0.955;1.181)** | 0.998 (0.979; 1.018)* | **0.981 (0.905; 1.064)*** | 1.024 (0.971; 1.080) |

Models: Generalized linear models with gamma distribution and log link for mean PD and mean CAL (reporting exponentiated beta coefficients with 95% confidence intervals), fractional response models for the percentage of sites with bleeding on probing and the percentage of sites with PD ≥3 mm (reporting exponentiated beta coefficients with 95% confidence intervals), and negative binomial regression models for incident tooth loss (reporting Incidence Rate Ratios with 95% confidence intervals). Models were adjusted for age (cont.), sex, school education, smoking, known or diagnosed diabetes mellitus, body mass index (cont.), tooth brushing frequency, use of interdental cleaning aids, use of powered tooth brushes, and serum magnesium. The unit for metabolites is normalised dimensionless intensities. Abbreviations: CAL, clinical attachment level; CI, confidence interval; IRR, Incidence Rate Ratio; N, number; PD, probing depth; RCS: restricted cubic spline term. * body mass index modelled as cubic splines with three knots; ** age modelled as cubic splines with three knots.

Appendix Table 4. Results from Inverse probability treatment weighted models and from regression-adjusted models using 11- and 16-year follow-up data of SHIP-START.

|  | Percentage of sites with bleeding on probing (N=953) | Mean PD  (N=953) | Percentage of sites  with PD ≥3 mm  (N=953) | Mean CAL  (N=953) |
| --- | --- | --- | --- | --- |
| **Inverse probability treatment weighted models** | | | | |
|  | Exp(beta) with 95% CI | Exp(beta) with 95% CI | Exp(beta) with 95% CI | Exp(beta) with 95% CI |
| Caffeine | 0.974 (0.907; 1.045)* | 0.999 (0.983; 1.016)* | 0.981 (0.931; 1.034)* | 1.002 (0.961; 1.044)* |
| Paraxanthine | **0.904 (0.819; 0.998)*** | RCS1: 1.042 (0.988; 1.099)  RCS2: 0.937 (0.882; 0.996) | 0.961 (0.900; 1.026) | **RCS1: 1.158 (1.011; 1.327)**  **RCS2: 0.821 (0.705; 0.957)** |
| Theobromine | RCS1: 0.850 (0.664; 1.087)  RCS2: 1.338 (0.926; 1.931) | **RCS1: 0.951 (0.908; 0.997)**  RCS2: 1.065 (0.999; 1.135) | **RCS1: 0.794 (0.670; 0.941)**  **RCS2: 1.346 (1.054; 1.720)** | RCS1: 0.892 (0.793; 1.004)  RCS2: 1.162 (0.981; 1.375) |
| Theophylline | 0.932 (0.846; 1.027)* | 0.991 (0.976; 1.007)* | 0.969 (0.906; 1.036)* | 0.995 (0.953; 1.039)* |
| **Regression-adjusted models** | | | | |
|  | Exp(beta) with 95% CI | Exp(beta) with 95% CI | Exp(beta) with 95% CI | Exp(beta) with 95% CI |
| Caffeine | 0.966 (0.908; 1.027)* | 0.997 (0.986; 1.007)* | 0.974 (0.932; 1.019)* | 0.999 (0.975; 1.024)* |
| Paraxanthine | **0.903 (0.823; 0.990)*** | RCS1: 1.034 (0.991; 1.080)  **RCS2: 0.945 (0.900; 0.993)*** | 0.959 (0.902; 1.019)* | **RCS1: 1.109 (1.006; 1.233)**  **RCS2: 0.864 (0.774; 0.965)*** |
| Theobromine | 1.026 (0.949; 1.108) | **RCS1: 0.951 (0.911; 0.992)**  **RCS2: 1.072 (1.010; 1.137)*** | **RCS1: 0.789 (0.670; 0.929)**  **RCS2: 1.384 (1.094; 1.751)** | **RCS1: 0.906 (0.827; 0.993)**  **RCS2: 1.157 (1.011; 1.325)*** |
| Theophylline | 0.936 (0.858; 1.022) | 0.994 (0.980; 1.008)* | 0.966 (0.909; 1.026)* | 1.005 (0.971; 1.040)* |

Models: Generalized linear models with gamma distribution and log link for mean PD and mean CAL (reporting exponentiated beta coefficients with 95% confidence intervals), fractional response models for the percentage of sites with bleeding on probing and the percentage of sites with PD ≥3 mm (reporting exponentiated beta coefficients with 95% confidence intervals), and negative binomial regression models for incident tooth loss (reporting Incidence Rate Ratios with 95% confidence intervals). Models were adjusted for age (cont.), sex, school education, smoking, known or diagnosed diabetes mellitus, body mass index (cont.), tooth brushing frequency, use of interdental cleaning aids, and use of powered tooth brushes. The unit for metabolites is normalised dimensionless intensities. Abbreviations: CAL, clinical attachment level; CI, confidence interval; IRR, Incidence Rate Ratio; N, number; PD, probing depth; RCS: restricted cubic spline term. * age modelled as restricted cubic spline with three knots

Appendix Table 5. Results from Inverse probability treatment weighted models and from regression-adjusted models using 11- and 16-year follow-up data of SHIP-START. With additional adjustment for baseline levels of serum magnesium.

|  | Percentage of sites with bleeding on probing (N=953) | Mean PD  (N=953) | Percentage of sites  with PD ≥3 mm  (N=953) | Mean CAL  (N=953) |
| --- | --- | --- | --- | --- |
| **Inverse probability treatment weighted models** | | | | |
|  | Exp(beta) with 95% CI | Exp(beta) with 95% CI | Exp(beta) with 95% CI | Exp(beta) with 95% CI |
| Caffeine | 0.973 (0.907; 1.044)* | 0.999 (0.983; 1.016)* | 0.981 (0.931; 1.034)* | 1.002 (0.962; 1.043)* |
| Paraxanthine | **0.902 (0.818; 0.995)*** | RCS1: 1.042 (0.987; 1.099)  RCS2: 0.938 (0.883; 0.996) | 0.962 (0.901; 1.026) | RCS1: 1.140 (0.993; 1.309)  **RCS2: 0.841 (0.719; 0.983)*** |
| Theobromine | RCS1: 0.849 (0.664; 1.087)  RCS2: 1.338 (0.926; 1.932) | **RCS1: 0.951 (0.908; 0.996)**  RCS2: 1.065 (0.999; 1.136) | **RCS1: 0.794 (0.670; 0.941)**  **RCS2: 1.346 (1.053; 1.720)** | RCS1: 0.892 (0.793; 1.004)  RCS2: 1.162 (0.981; 1.376) |
| Theophylline | 0.927 (0.842; 1.019)* | 0.992 (0.977; 1.007)* | 0.968 (0.905; 1.035)* | 0.999 (0.957; 1.044)* |
| **Regression-adjusted models** | | | | |
|  | Exp(beta) with 95% CI | Exp(beta) with 95% CI | Exp(beta) with 95% CI | Exp(beta) with 95% CI |
| Caffeine | 0.966 (0.908; 1.027)* | 0.997 (0.986; 1.007)* | 0.974 (0.932; 1.019)* | 0.999 (0.975; 1.024)* |
| Paraxanthine | **0.901 (0.822; 0.987)*** | RCS1: 1.035 (0.991;1.081)  **RCS2: 0.944 (0.898; 0.992)*** | 0.957 (0.901; 1.017)* | **RSC1: 1.109 (1.006; 1.222)**  **RCS2: 0.864 (0.774; 0.965)*** |
| Theobromine | 1.025 (0.948; 1.108) | **RCS1: 0.948 (0.909; 0.989)**  **RCS2: 1.075 (1.014; 1.140)*** | **RCS1: 0.783 (0.665; 0.922)**  **RCS2: 1.398 (1.107; 1.767)** | **RCS1: 0.906 (0.827; 0.992)**  **RCS2: 1.157 (1.011; 1.325)*** |
| Theophylline | 0.933 (0.856; 1.018)* | 0.993 (0.979; 1.008)* | 0.964 (0.907; 1.024)* | 1.005 (0.971; 1.041)* |

Models: Generalized linear models with gamma distribution and log link for mean PD and mean CAL (reporting exponentiated beta coefficients with 95% confidence intervals), fractional response models for the percentage of sites with bleeding on probing and the percentage of sites with PD ≥3 mm (reporting exponentiated beta coefficients with 95% confidence intervals), and negative binomial regression models for incident tooth loss (reporting Incidence Rate Ratios with 95% confidence intervals). Models were adjusted for age (cont.), sex, school education, smoking, known or diagnosed diabetes mellitus, body mass index (cont.), tooth brushing frequency, use of interdental cleaning aids, use of powered tooth brushes, and serum magnesium. The unit for metabolites is normalised dimensionless intensities. Abbreviations: CAL, clinical attachment level; CI, confidence interval; IRR, Incidence Rate Ratio; N, number; PD, probing depth; RCS: restricted cubic spline term. * age modelled as restricted cubic spline with three knots

Appendix Table 6. Results from inverse probability treatment weighted mixed models using 11-, 16- and 21-year follow-up data of SHIP-START.

|  | Percentage of sites with bleeding on probing  (N=953 groups/2542 obs.) | Mean PD  (953 groups/2542 obs.) | Percentage of sites with PD ≥3 mm  (953 groups/2542 obs.) | Mean CAL  (953 groups/2533 obs.) |
| --- | --- | --- | --- | --- |
|  | Beta with 95% CI | Exp(beta) with 95% CI | Beta with 95% CI | Exp(beta) with 95% CI |
| Caffeine | -0.016 (-0.985; 0.952)* | 1.001 (0.991; 1.011)* | 0.071 (-0.964; 1.10)* | 1.014 (0.986; 1.043)* |
| Paraxanthine | -0.090 (-1.05; 0.874)* | **RCS1: 1.060 (1.019; 1.102)**  **RCS2: 0.934 (0.891; 0.979)*** | **RCS1: 5.12 (0.965; 9.28)**  **RCS2: -5.97 (-11.0; -0.983)*** | **RCS1: 1.223 (1.094;1.366)**  **RCS2: 0.825 (0.723; 0.943)*** |
| Theobromine | -0.145 (-1.18; 0.892) | **RCS1: 0.942 (0.911; 0.975)**  **RCS2: 1.065 (1.013; 1.119)** | **RCS1: -6.574 (-10.2; -2.90)**  **RCS2: 7.190 (1.89; 12.5)** | **RCS1: 0.875 (0.793; 0.967)**  **RCS2: 1.177 (1.020; 1.358)** |
| Theophylline | -0.153 (-1.51; 1.20)* | 1.002 (0.988; 1.015)* | 0.163 (-1.27; 1.60)* | 1.037 (0.998; 1.078)* |
| **With additional adjustment for baseline levels of serum magnesium** | | | | |
|  | Beta with 95% CI | Exp(beta) with 95% CI | Beta with 95% CI | Exp(beta) with 95% CI |
| Caffeine | -0.020 (-0.987; 0.948)* | 1.001 (0.991; 1.011)* | 0.071 (-0.963; 1.10)* | 1.014 (0.986; 1.043)* |
| Paraxanthine | -0.071 (-1.50; 1.36)* | **RCS1: 1.060 (1.020; 1.102)**  **RCS2: 0.934 (0.891; 0.978)*** | **RCS1: 5.13 (0.979; 9.29)**  **RCS2: -5.99 (-11.0; -1.00)*** | **RCS1: 1.223 (1.095; 1.366)**  **RCS2: 0.824 (0.722; 0.942)*** |
| Theobromine | -0.146 (-1.18; 0.891) | **RCS1: 0.942 (0.911; 0.975)**  **RCS2: 1.065 (1.013; 1.119)** | **RCS1: -6.57 (-10.2; -2.90)**  **RCS2: 7.19 (1.88; 12.5)** | **RSC1: 0.876 (0.794; 0.967)**  **RCS2: 1.177 (1.020; 1.358)** |
| Theophylline | -0.169 (-1.53; 1.19)* | 1.002 (0.989; 1.015) | 0.165 (-1.27; 1.60)* | 1.038 (0.999; 1.079)* |

Models: Generalized linear mixed regression models with gamma distribution and log link for mean PD and mean CAL (reporting exponentiated beta coefficients with 95% confidence intervals), linear mixed regression models for the percentage of sites with bleeding on probing and the percentage of sites with PD ≥3 mm (reporting beta coefficients with 95% confidence intervals), and negative binomial mixed regression models for the number of missing teeth (reporting Incidence Rate Ratios with 95% confidence intervals). Models were adjusted for age (cont.), sex, school education, smoking, known or diagnosed diabetes mellitus, body mass index (cont.), tooth brushing frequency, use of interdental cleaning aids, and use of powered tooth brushes. The unit for metabolites is normalised dimensionless intensities. Abbreviations: CAL, clinical attachment level; CI, confidence interval; IRR, Incidence Rate Ratio; N, number; PD, probing depth; RCS: restricted cubic spline term. * age modelled as restricted cubic spline with three knots.


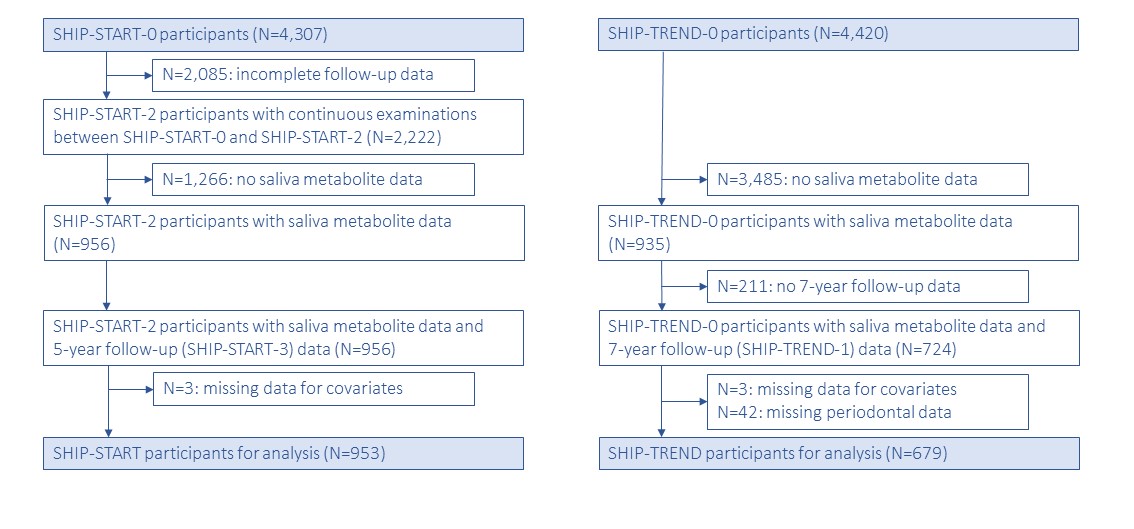
Appendix Figure 1. Flowchart for the selection of SHIP-START and SHIP-TREND participants for longitudinal analyses.
